# Nicotine Reward and Affective Nicotine Withdrawal Signs Are Attenuated in Calcium/Calmodulin-Dependent Protein Kinase IV Knockout Mice

**DOI:** 10.1371/journal.pone.0051154

**Published:** 2012-11-30

**Authors:** Kia J. Jackson, Sarah S. Sanjakdar, Xiangning Chen, M. Imad Damaj

**Affiliations:** 1 Department of Psychiatry, Virginia Commonwealth University, Richmond, Virginia, United States of America; 2 Department of Pharmacology and Toxicology, Virginia Commonwealth University, Richmond, Virginia, United States of America; IGBMC/ICS, France

## Abstract

The influx of Ca^2+^ through calcium-permeable nicotinic acetylcholine receptors (nAChRs) leads to activation of various downstream processes that may be relevant to nicotine-mediated behaviors. The calcium activated protein, calcium/calmodulin-dependent protein kinase IV (CaMKIV) phosphorylates the downstream transcription factor cyclic AMP response element binding protein (CREB), which mediates nicotine responses; however the role of CaMKIV in nicotine dependence is unknown. Given the proposed role of CaMKIV in CREB activation, we hypothesized that CaMKIV might be a crucial molecular component in the development of nicotine dependence. Using male CaMKIV genetically modified mice, we found that nicotine reward is attenuated in CaMKIV knockout (−/−) mice, but cocaine reward is enhanced in these mice. CaMKIV protein levels were also increased in the nucleus accumbens of C57Bl/6 mice after nicotine reward. In a nicotine withdrawal assessment, anxiety-related behavior, but not somatic signs or the hyperalgesia response are attenuated in CaMKIV −/− mice. To complement our animal studies, we also conducted a human genetic association analysis and found that variants in the CaMKIV gene are associated with a protective effect against nicotine dependence. Taken together, our results support an important role for CaMKIV in nicotine reward, and suggest that CaMKIV has opposing roles in nicotine and cocaine reward. Further, CaMKIV mediates affective, but not physical nicotine withdrawal signs, and has a protective effect against nicotine dependence in human genetic association studies. These findings further indicate the importance of calcium-dependent mechanisms in mediating behaviors associated with drugs of abuse.

## Introduction

Activation of neuronal nicotinic receptors (nAChRs) by nicotine results in increased permeability to Na^+^ and Ca^2+^ [1]. Permeability to Ca^2+^ allows for several long-term and short-term Ca^2+^-dependent processes to occur, which result in neurotransmitter release, synaptic plasticity, and upregulation of genes required for receptor synthesis [2]. These processes are thought to be important in mediating acute and chronic effects of nicotine.

Some of the targets of nicotine-induced calcium influx are Ca^2+^/calmodulin-dependent kinases, such as Ca^2+^/calmodulin-dependent kinase II (CaMKII) and Ca^2+^/calmodulin-dependent kinase IV (CaMKIV). CaMKII, a serine threonine specific kinase regulated by a Ca^2+^/calmodulin complex and abundant in brain tissue, is necessary for LTP induction, enhances synaptic transmission, and mediates neurotransmitter secretion [2]. Unlike CaMKII, CaMKIV is not as ubiquitously expressed, and is primarily found in the hippocampus, cerebellum, and amygdala, as well as in spleen, thymus, and testis [3], [4], [5], [6], [7]. CaMKIV is also located mainly in the neuronal nuclei and is involved in the regulation of activity-triggered gene expression [8]. One specific transcription factor CaMKIV regulates is cyclic AMP response element binding protein (CREB) [9], which is implicated in cocaine, morphine, and nicotine reward [10], [11]. In addition, CaMKIV contributes to the activation of CREB in various memory-related areas, such as the amygdala and hippocampus [12]. Using CaMKIV knockout (−/−) mice, Ko et al. [13] provided evidence for a role of CaMKIV in the development of opioid analgesic tolerance but not physical dependence. Interestingly, mice lacking CaMKIV in dopaminoceptive neurons show increased sensitivity to cocaine locomotor sensitization and conditioned place preference (CPP), a reward-associated behavior, in a CREB-independent manner [14]. However, the role of CaMKIV in nicotine’s effects is unknown. Recent studies showed that CREB activation in the brain [15], and more specifically, in the NAc, are critical for nicotine-induced CPP [16]. Furthermore, changes in CREB activity in the VTA and NAc of mice were shown to accompany withdrawal in nicotine-dependent mice [17]. Given the proposed role of CaMKIV in CREB activation, we hypothesized that CaMKIV might be a crucial molecular component in the development of nicotine dependence. To test this hypothesis, we used CaMKIV −/− mice to elucidate the contribution of CaMKIV to nicotine dependence-like behaviors. We first assessed in the CaMKIV −/−, heterozygote (+/−), and wild-type (+/+) mice, the rewarding effects of nicotine using the CPP test. We then measured changes in CaMKIV levels in the NAc after exposure to nicotine in the CPP test. Because CREB activity is also altered after cocaine administration [10], we tested the specificity of CaMKIV’s role in nicotine behaviors by measuring cocaine CPP in genetically modified CaMKIV mice. Additionally, we assessed physical (somatic signs and hyperalgesia) and affective (anxiety-related behavior) nicotine withdrawal signs in CaMKIV mice. Finally, to assess the relevance of these behavioral changes to nicotine dependence in humans, we conducted a genetic association analysis to determine if polymorphisms in the human CaMKIV gene are associated with nicotine dependence. Our findings support a significant role for CaMKIV in nicotine and cocaine reward, as well as nicotine withdrawal, and emphasize the importance of calcium-dependent mechanisms in mediating drug-induced behaviors.

## Materials and Methods

### Ethics Statement

For studies involving animal subjects, mice were group-housed in a 21°C humidity-controlled Association for Assessment and Accreditation of Laboratory Animal Care-approved animal care facility with ad libitum access to food and water. Experiments were performed during the light cycle and were approved by the Institutional Animal Care and Use Committee of Virginia Commonwealth University. Experiments were designed so that the minimum number of animals necessary to obtain concrete results were used. Animals were sacrificed by CO_2_ asphyxiation immediately following the withdrawal assessment to alleviate any suffering associated with the experiment. For all other studies, animals were sacrificed by CO_2_ asphyxiation the evening or morning following the experiment unless otherwise noted.

All datasets for human genetic association analysis were obtained via the NCBI dbGAP website http://www.ncbi.nlm.nih.gov/gap under a protocol approved by the Virginia Commonwealth University Institutional Review Board and the National Institutes of Health.

### Animals

Male C57Bl/6 mice and male and female breeders null for the CaMKIV gene and +/+ littermates were purchased from Jackson Laboratories (Bar Harbor, ME, USA). CaMKIV −/− and their +/+ littermates were bred in an animal care facility at Virginia Commonwealth University. For all experiments, mice were backcrossed at least 8 to 10 generations. Mutant and +/+ mice were obtained from crossing +/− mice. This breeding scheme controlled for any irregularities that might occur with crossing solely mutant animals. Animals were 8–10 weeks of age at the start of all studies.

### Drugs

(−)-Nicotine hydrogen tartrate salt and mecamylamine hydrochloride, were purchased from Sigma-Aldrich Inc. (St. Louis, MO, USA). Cocaine was obtained from the National Institute on Drug Abuse (Bethesda, MD, USA). All drugs were dissolved in physiological saline (0.9% sodium chloride) at a volume of 10 ml/kg body weight. Nicotine and mecamylamine were administered subcutaneously (s.c.) and cocaine was administered intraperitoneally (i.p.). Doses are expressed as the free base of the drug.

### Nicotine and Cocaine CPP

Nicotine CPP (n = 8−15 per group) and cocaine CPP (n = 6−7 per group) were conducted on independent groups of CaMKIV genetically modified mice using an unbiased design as previously described by Kota et al [18]. In brief, mice were handled for three days prior to initiation of CPP testing. The CPP apparatus consisted of a three-chambered box with a white compartment, a black compartment, and a center grey compartment. The black and white compartments also had different floor textures to help the mice further differentiate between the two environments. On day 1, mice were placed in the grey center compartment for a 5 min habituation period, followed by a 15 min test period to determine baseline responses. A pre-preference score was recorded and used to randomly pair each mouse with either the black or white compartment. Drug-paired sides were randomized so that an even number of mice received drug on the black and white side. Over the next 3 days, mice were conditioned twice a day, once in the morning (7–9 am) and once in the afternoon (2–4 pm), for 20 min with the saline group receiving saline on both sides of the boxes and drug groups receiving nicotine (0.5 mg/kg, s.c.) or cocaine (2.5 mg/kg, i.p.) on one side of the box and saline on the opposite side. Animals in the drug group received drug each day. Injections were counterbalanced so that some mice received drug in the morning, others in the evening. Test day (Day 5) was a drug free day, and animals were placed into the chambers similarly to day 1 procedure. Locomotor activity counts and time spent on each side were recorded, and data were expressed as time spent on the drug-paired side post-conditioning minus time spent on the drug-paired side pre-conditioning. A positive number indicated a preference for the drug-paired side, whereas a negative number indicated an aversion to the drug-paired side. A number at or near zero indicated no preference for either side.

### CaMKIV Western Blot Analysis

Nicotine CPP (0.5 mg/kg) was conducted in a separate group of male C57Bl/6 mice (n = 3−5 per group). Mice were sacrificed by cervical dislocation 5 minutes after removal from the CPP chambers on test day. Brains were rapidly removed and sliced into 1 mm thick sections using a mouse brain matrix (Braintree Scientific Co., Braintree, MA, USA) on ice. The NAc, consisting of both the shell and core divisions, was identified using a stereotaxic atlas [19] dissected from the appropriate section (approximate coordinates NAc: Bregma 1.10 mm), and homogenized immediately in cold extraction buffer containing 50 mM Tris, 1% SDS, 1 mM PMSF, 1 mM EDTA, 5 mM EGTA, 1 mM Na^+^ orthovanadate, 20 µg/ml leupeptin, 10 µg/ml aprotinin, and 1 µM okadaic acid. Protein concentrations were determined using the Bradford assay. 30 µg of protein were incubated with 6X blue gel loading dye (New England Biolabs, Ipswich, MA, USA), and heated for 5 minutes at 95°C. Samples were then separated by SDS-polyacrylamide gel electrophoresis on a 10% TRIS-HCl gel and subjected to immunoblotting. Non-specific protein was blocked in 5% milk solution in TBS-T for 1 hour at room temperature. Primary antibodies for CaMKIV (1∶1000; Cell Signaling, Danvers, MA, USA) and α-tubulin antibody (1∶5000; Upstate, Temecula, CA, USA) were incubated overnight at 4°C. Secondary antibodies (1∶5000; LiCor Biosciences, Inc., Lincoln, NE, USA) were incubated for 1 hour at room temperature the next day. Bound antibody was detected using the LiCor Odyssey Infrared Imaging System (LiCor Biosciences, Inc., Lincoln, NE, USA). CaMKIV bands were detected at 63 kDa and α-tubulin bands were detected at 55 kDa. Blots were analyzed by taking the ratio of protein:αtubulin.

### Mecamylamine-precipitated Nicotine Withdrawal Assessment

Withdrawal studies were carried out as previously described by Jackson et al [20]. In brief, mice were anesthetized with sodium pentobarbital (45 mg/kg i.p.) and implanted with Alzet osmotic minipumps [model 2002 (14 days); Durect Corporation, Cupertino, CA] filled with (-)-nicotine (36 mg/kg/day) or saline solution for 14 days. The concentration of nicotine was adjusted according to animal weight and minipump flow rate. On the morning of day 15, mice (n = 4−6 per group) were injected s.c. with vehicle or 2 mg/kg mecamylamine 10 min before testing. Mice were first evaluated for 5 min in the plus maze test for anxiety-related behavior, followed by a 20 minute somatic sign observation period, during which mice were observed for paw and body tremors, head shakes, backing, jumps, curls, and ptosis. Hyperalgesia was evaluated immediately after the somatic sign observation period. This specific testing sequence is carried out based on prior studies by Jackson et al [20] showing that this order of testing results in the lowest within-group variability while producing consistent results. The number of arm crosses was also counted in the plus maze as a measure of locomotor activity.

### CaMKIV Human Genetic Association Study

**Figure 1:**
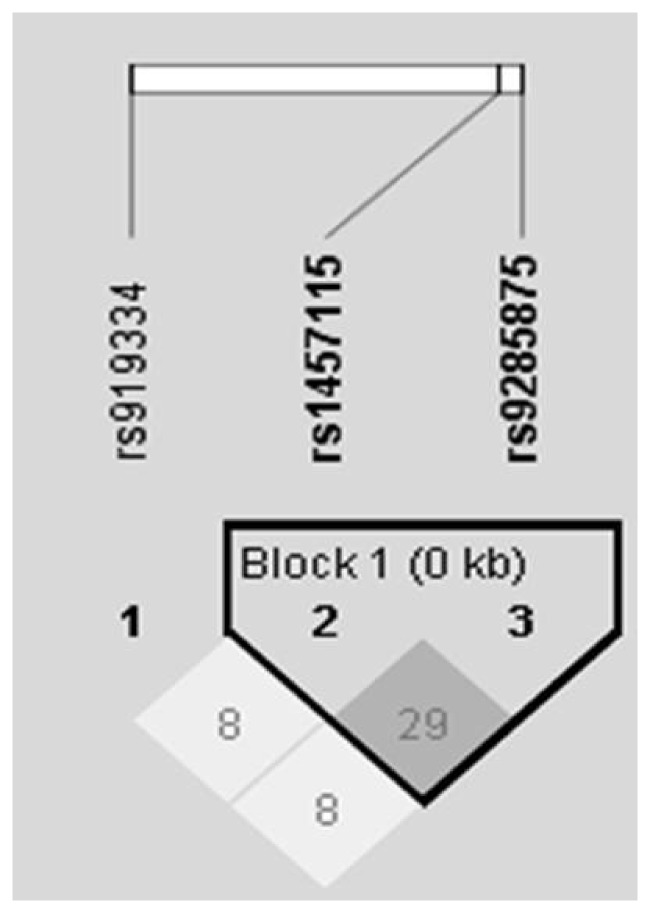
Linkage disequilibrium block containing r-squared values for the MGS and SAGE datasets combined. Values within the squares represent r-squared values.

Male and female European-American subjects from the Molecular Genetics of Schizophrenia (MGS) genome wide association study and the Study of Addition: Genetics and Environment (SAGE) dataset were used to assess the possible association of polymorphisms in the CaMKIV gene with nicotine dependence. In the MGS study, only smoking data from control subjects were used in the analysis, totaling 1,819 subjects with genotype and phenotype data available. The SAGE study is comprised of three independent studies: the Collaborative Genetic Study of Nicotine Dependence (COGEND), the Collaborative Study on the Genetics of Alcoholism (COGA), and the Family Study of Cocaine Dependence (FSCD). Smoking data from each of the three studies were used in the analysis, totaling 2,150 subjects with genotype and phenotype data available for analysis. The Fagerström Test for Nicotine Dependence (FTND) scores, a categorized number of cigarettes smoked per day (numCIG) phenotype (grouped as 10 or less, 11–20, 21–30, 31 or more), and two withdrawal assessment questions from the FTND questionnaire (FTND question 1- How soon after smoking do you smoke your first cigarette?, FTND question 5- Do you smoke more cigarettes in the morning than the rest of the day?) were used as phenotypic measures of nicotine dependence. The markers rs919334, rs1457115, and rs9285875, located in the promoter region of the human CaMKIV gene, were recently found to be associated with risk for cocaine dependence [14], thus, we chose these makers to test for an association with nicotine dependence. A linkage disequilibrium block with r-squared values for the three markers is shown in Figure 1. The marker rs919334 was imputed in the MGS dataset, as it was not initially genotyped. The imputation error rate was approximately 6%, indicating good imputation quality. To increase the power of our analysis, a meta-analysis was conducted using results from both datasets (n = 3,969).

### Statistical Analysis

Statistical analyses were performed using a two-way analysis of variance test for all behavioral studies with treatment and genotype as between-subject factors. One-way analysis of variance was used for the CaMKIV western blot analysis. All differences were considered significant at p<0.05, with a Student Newman-Kuels post-hoc test used to further analyze significant results. Statistical analysis for genetic association studies was performed using the PLINK software [21]. FTND score, numCIG, and FTND question 1 (FTND1) were treated as continuous traits and were analyzed by linear regression. FTND question 5 (FTND5) was treated as a dichotomized variable and was analyzed using logistic regression. Age, sex, and principal components to control for population stratification within the sample were used as covariates. In the SAGE dataset, study (COGA,COGEND, or FSCD) was also used as a covariate.

Imputation in the MGS dataset was conducted using fastPHASE [22], with the Hapmap CEU (Utah residents with Northern and Western European ancestry) dataset as a reference panel. The GWAMA program [23] was used for a meta-analysis of the results from both datasets. Cochrane’s Q statistic p-values were calculated to measure between-study heterogeneity. For the haplotype analysis, the MGS and SAGE datasets were combined using the PLINK software, and principal components were generated in the combined dataset using the EIGENSTRAT software [24] to control for population stratification between dataset. Haplotype analysis was conducted using the proxy association feature in the PLINK software with rs919334 as the reference marker, and using age, sex, and principal components as covariates. A Bonferroni correction (p-value x no. of markers x no. of phenotypes x no. of datasets) was applied to correct for multiple testing in the meta-analysis. Uncorrected p-values are reported in the tables.

## Results

### Nicotine does not Induce a Significant CPP in CaMKIV −/− Mice

**Figure 2:**
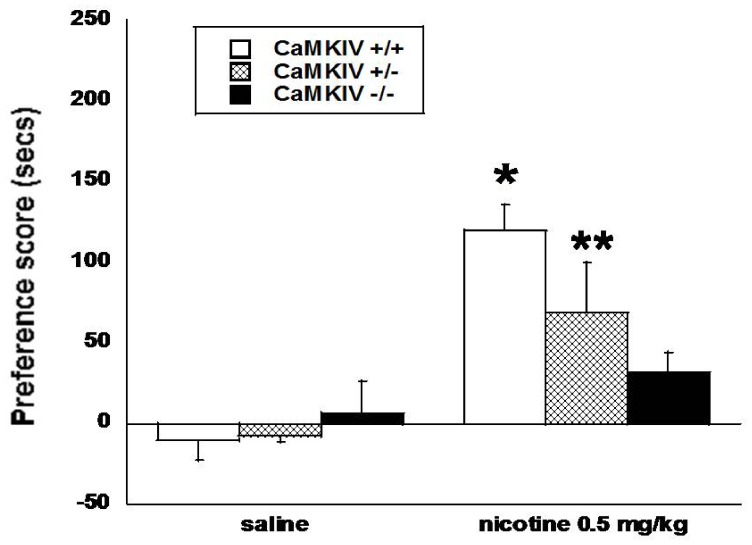
Nicotine CPP is attenuated in CaMKIV −/− mice. Male CaMKIV +/+ and +/− mice develop a significant preference for nicotine (0.5 mg/kg, s.c.); however, this effect is eliminated in CaMKIV −/− mice. *denotes p<0.05 vs. corresponding saline groups and CaMKIV −/− nicotine group. **denotes p<0.05 vs. corresponding saline groups. Each point represents the mean ± SEM for 8–15 mice per group. The y-axis represents the preference score (drug-paired side test day – drug paired side baseline) in seconds. The x-axis represents the treatment.

Male CaMKIV genetically modified mice were conditioned with 0.5 mg/kg nicotine for three days and preference scores were assessed. There were significant main effects of treatment [F(_1,59_) = 21.70, p<0.0001], but no main effects of genotype, suggesting no significant difference between genotypes in this paradigm; however, a significant treatment x genotype interaction [F(_2,59_) = 4.03, p<0.05] was detected. Results show that nicotine induced a significant CPP in CaMKIV +/+ and +/− mice; however, failed to produce a significant CPP response in CaMKIV −/− mice (Figure 2). CaMKIV −/− mice did not differ from saline treated CaMKIV +/+, +/−, or −/− counterparts after nicotine conditioning. No significant differences in baseline activity were observed between genotypes.

### CaMKIV Protein Level is Significantly Increased in the NAc After Nicotine CPP

**Figure 3:**
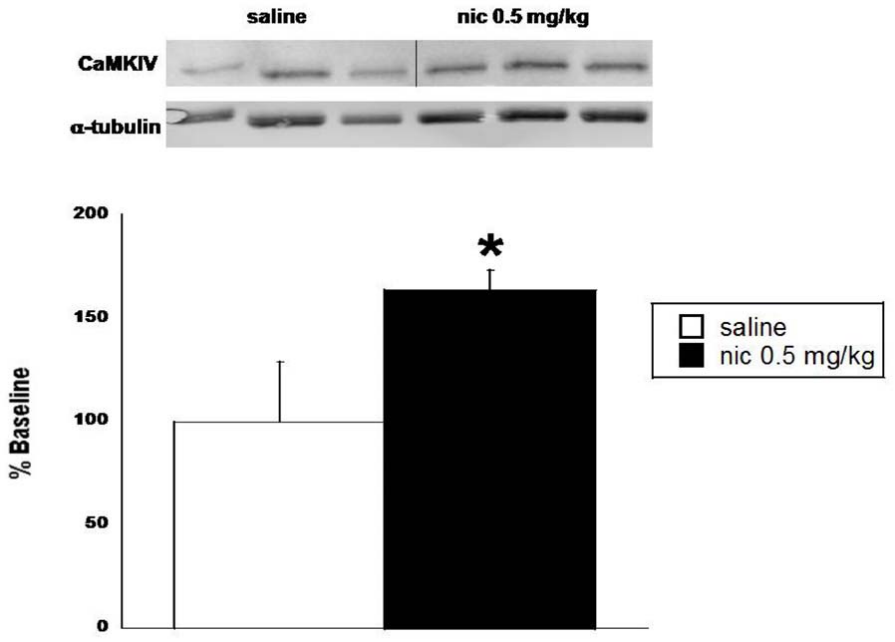
CaMKIV protein levels are increased after nicotine CPP. Brains were removed and the NAc was dissected from male C57Bl/6 mice within 5 min after removal from CPP chambers on test day. Conditioning with nicotine (0.5 mg/kg, s.c.) induced a significant increase in CaMKIV protein level in the NAc after CPP. A representative western blot is shown above the graph. Each point represents the mean ± SEM for 3–5 mice per group. The y-axis represents the percentage of saline baseline. *denotes p<0.05 vs. saline group.

Male C57Bl/6 mice were conditioned in the nicotine CPP paradigm (data not shown), and the NAc was dissected immediately after removal from the CPP chambers on test day to measure CaMKIV protein level after nicotine CPP. Nicotine CPP induced a significant increase in CaMKIV protein level in the NAc of mice previously conditioned with nicotine [Figure 3; F(_1,6_) = 6.73, p<0.05]. Saline conditioning had no significant effect on CaMKIV levels.

### Cocaine CPP is Significantly Enhanced in CaMKIV −/− Mice

**Figure 4:**
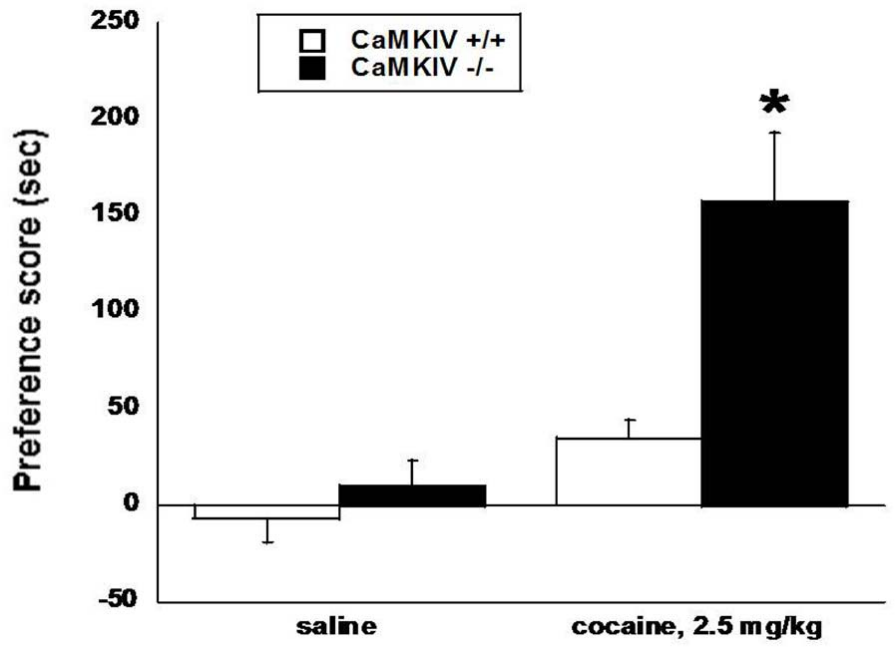
Cocaine CPP is enhanced in CaMKIV −/− mice. Male CaMKIV +/+ mice do not develop a significant cocaine CPP after conditioning with a low cocaine dose (2.5 mg/kg, i.p.) that does not produce a significant cocaine CPP in +/+ mice; however, CaMKIV −/− mice develop a robust CPP after cocaine conditioning. Each point represents the mean ± SEM for 6–7 mice per group. *denotes p<0.05 vs. corresponding saline groups and CaMKIV +/+ cocaine-treated mice. The y-axis represents the preference score (drug-paired side test day – drug paired side baseline) in seconds. The x-axis represents the treatment.

Male CaMKIV +/+, +/−, and −/− mice were conditioned with a low dose of cocaine (2.5 mg/kg, i.p.) that does not produce a significant cocaine CPP in +/+ mice. There were significant main effects of treatment [F(_1,22_) = 23.40, p<0.0001], genotype [F(_1,22_) = 7.34, p<0.05], and a significant genotype x treatment interaction [F(_1,22_)] = 12.82, p<0.05). The dose of cocaine did not produce a significant CPP in CaMKIV +/+ or +/− mice [CaMKIV +/− CPP scores: 40±14.31 sec]; however, a robust CPP was observed in CaMKIV −/− mice (Figure 4). There were no significant differences in saline baseline between genotypes.

### Affective, but not Physical Nicotine Withdrawal Signs are Altered in CaMKIV −/− Mice

**Figure 5:**
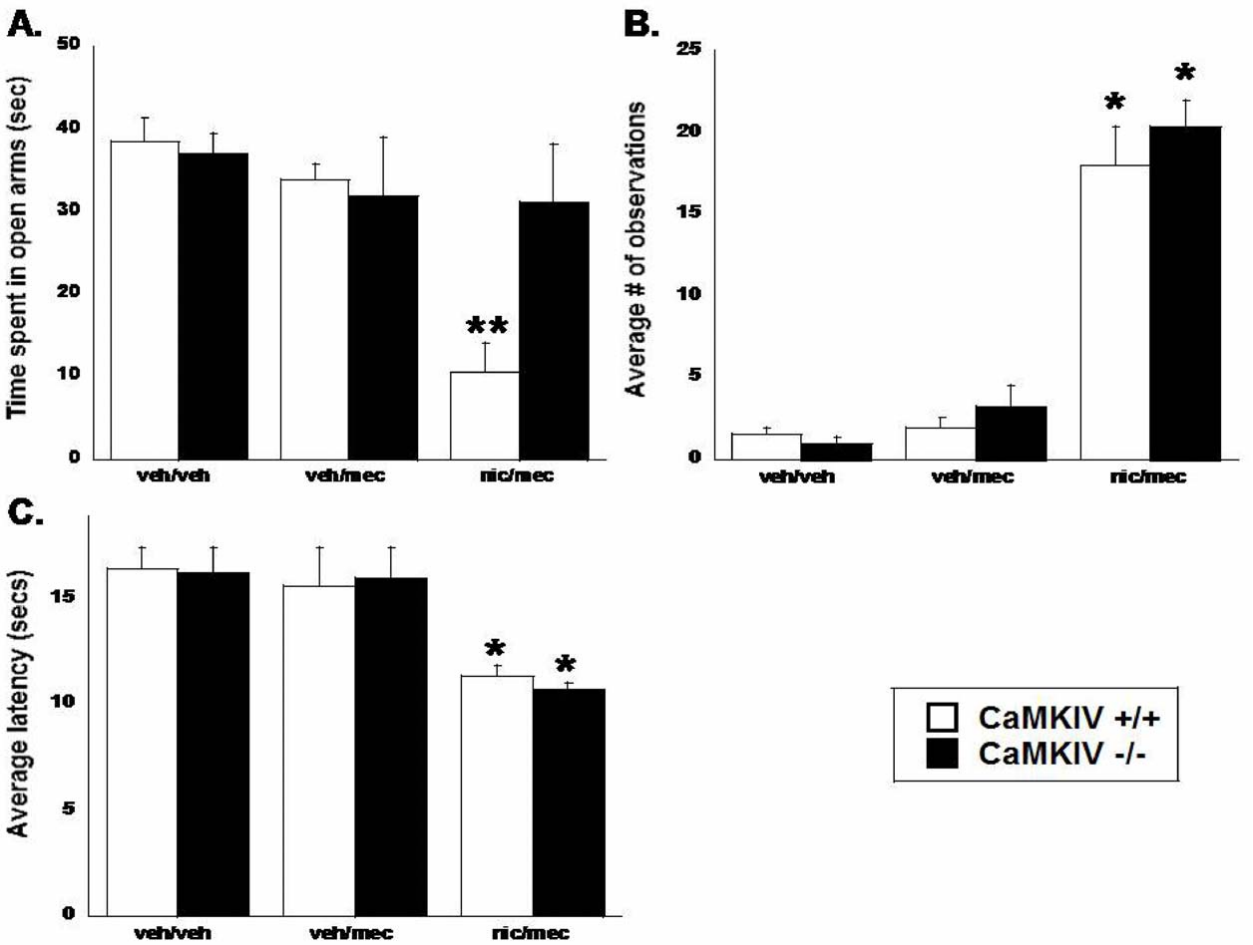
CaMKIV mediates affective, but not physical nicotine withdrawal signs. A. Nicotine-infused CaMKIV +/+ mice show significant anxiety-like behavior after mecamylamine (2 mg/kg, s.c. at day 15) treatment in chronically infused nicotine mice (36 mg/kg/day for 14 days). Withdrawal-induced anxiety-like behaviors are reduced in nicotine-infused CaMKIV −/− mice. The y axis represents the time spent in the open arms of the plus maze in seconds. **B**. Somatic nicotine withdrawal signs are not significantly attenuated in nicotine-infused CaMKIV −/− mice compared to +/+ counterparts. The y-axis represents the average number of somatic signs observed during the 20 min session. **C.** The nicotine withdrawal-induced hyperalgesia response is observed similarly in CaMKIV +/+ and −/− mice. The y-axis represents the average hot plate latency in seconds. Each point represents the mean ± SEM of 4–6 mice per group. *denotes p<0.05 vs. vehicle groups. **denotes p<0.05 vs. vehicle and CaMKIV −/− nic/mec group. Veh, vehicle; mec, mecamylamine; nic, nicotine.

Male CaMKIV +/+ and −/− mice were chronically infused with saline or nicotine (36 mg/kg/day) through osmotic minipumps for 14 days and treated with mecamylamine (2 mg/kg, s.c.) on day 15 to precipitate nicotine withdrawal signs. Results are shown in Figure 4. Main effects of treatment were observed in each nicotine withdrawal test. In nicotine infused CaMKIV +/+ mice, mecamylamine precipitated significant anxiety-related behavior [F(_2,24_) = 10.06, p<0.001], indicated by a decrease in the amount of time spent in the open arms of the plus maze (Figure 5A), increased somatic signs [F(_2,24_) = 120.16, p<0.0001; Figure 5B], and a significant hyperalgesia response [F(_2,24_) = 10.29, p<0.001], indicated by a decreased latency in the hot plate test (Figure 5C). For the plus maze test, there was also a significant genotype x treatment interaction [F(_2,24_) = 5.48, p<0.05]. While mecamylamine induced a significant anxiety-related response in nicotine-infused CaMKIV +/+ mice, mecamylamine failed to precipitate such a response in CaMKIV −/− mice (Figure 5A). In contrast, mecamylamine precipitated significant somatic signs (Figure 5B) and hyperalgesia (Figure 5C) in nicotine-infused CaMKIV −/− mice, similar to that observed in +/+ mice. Results from individual somatic signs are shown in Table 1. Interestingly, paw tremors were found to be significantly more intense in nicotine treated CamKIV −/− mice compared to +/+ counterparts [significant main effects of treatment: F(_2,24_) = 112.04, p<0.0001; significant main effects of genotype: F(_2,24_) = 19.60, p<0.05; significant treatment x genotype interaction: F(_2,24_) = 11.59, p<0.0005], though this had no significant impact on the total average number of somatic signs, as CaMKIV −/− mice did not differ from +/+ for total somatic signs. There were no significant differences between saline CaMKIV +/+ and −/− mice in these assessments, and no significant difference in the number of arm crosses between genotypes in the plus maze test.

**Table 1:** Average number of individual somatic signs assessed in CaMKIV +/+ and −/− mice.

Groups	Paw tremor	Body tremor	Head shakes	Backing	Others
veh/veh CaMKIV +/+	1.2±0.2	0	0.4±0.2	0	0
veh/mec CaMKIV +/+	1.2±0.7	0	0.3±0.3	0.5±0.3	0
nic/mec CaMKIV +/+	7.2±1.1*	2±0.6*	5.8±2.2*	2.8±1.0*	0.4±0.2*
veh/veh CaMKIV −/−	0.6±0.4	0	0.4±0.2	0	0
veh/mec CaMKIV −/−	1.3±0.5	0	1.75±1.1	0.25±0.3	0
nic/mec CaMKIV −/−	12.6±0.8* ^+^	2±0.5*	2.6±0.2	2.8±0.3*	0.4±0.4*

Each point represents the mean ± SEM of 4–6 mice per group. The heading “others” refers to jumps, curls, and ptosis.

* denotes p<0.05 vs. corresponding vehicle groups. + denotes p<0.05 vs. CamKIV nic/mec +/+ mice. veh, vehicle; nic, nicotine; mec, mecamylamine.

### Variants in CaMKIV are Associated with Nicotine Dependence Phenotypes

Our animal data suggests that CaMKIV is involved in nicotine-mediated behaviors. To support these findings, we tested the markers rs919334, rs1457115, and rs9285875**,** located within the promoter region of the CaMKIV gene, for an association with nicotine dependence in two independent datasets and conducted a meta-analysis of the results. Results are shown in Table 2. Cochrane’s Q statistic p-values were significant for the rs919334 marker in the FTND phenotype (p = 0.02) and marginal for the FTND1 phenotype (p = 0.06). Thus, a random effects meta-analysis was used to analyze the data to account for possible heterogeneity between datasets. The marker rs919334 was significantly associated with a protective effect in the FTND analysis, suggesting an association with lower FTND scores. Interestingly, the marker rs9285875 was associated with risk for higher FTND scores. Neither marker survived correction for multiple testing.

**Table 2:** Variants in the CaMKIV gene are associated with nicotine dependence.

Phenotype	SNP	Allele	P-value	Beta/OR	Q p-value
**FTND**	**rs919334**	C	**0.01**	**−0.185**	**0.41**
	rs1457115	A	0.53	−0.044	0.51
	**rs9285875**	T	**0.04**	**0.163**	**0.82**
**numCIG**	rs919334	C	0.17	−0.034	0.20
	rs1457115	A	0.95	−0.001	0.45
	rs9285875	T	0.43	0.021	0.88
**FTND1**	rs919334	C	0.14	−0.045	0.06
	rs1457115	A	0.11	0.046	0.86
	rs9285875	T	0.63	−0.015	0.56
**FTND5**	rs919334	C	0.33	0.94	*0.02*
	rs1457115	A	0.50	1.04	0.72
	rs9285875	T	0.87	1.01	0.08

Human genetic association meta-analysis using two datasets (SAGE and MGS, n = 3,969). The variant rs919334 is associated with a protective effect in the FTND score analysis, while rs9285875 is associated with risk for higher FTND scores. The table contains uncorrected p-values, where significant p- values are bolded and underlined. No markers survived correction for multiple testing. Significant Q p-values are italicized. SNP, single nucleotide polymorphism; the Allele column represents the minor allele; Q p-values represent Cochrane’s Q-statistic p-values.

Based on these findings, we conducted a haplotype analysis using the combined MGS and SAGE dataset and markers [rs919334-rs1457115-rs9285875] (Table 3). We found significant multi-marker combinations for FTND score, numCIG, and FTND5. The C-G-T haplotype consisting of both the protective rs919334 allele and the risk rs9285875 allele represented a significant protective haplotype for FTND score, numCIG (corrected p = 0.002) and FTND5 (corrected p = 0.0002). Similar protective effects were observed with the T-G-C haplotype for FTND, numCIG (corrected p = 0.005), and FTND5 (corrected p = 0.0005), which consists of the major alleles from each marker, and for C-A-C for FTND scores. Alternatively, the T-G-T haplotype consisting of the minor risk allele for rs9285875 confers risk for higher FTND scores, smoking more cigarettes per day (numCIG), and smoking more in the morning than any other time of day (FTND5), though none of these associations survived correction for multiple testing.

**Table 3:** Haplotype analysis of the three CaMKIV variants.

Phenotype	Haplotype	P-value	Beta/OR
**FTND**	**CGT (0.03)**	**0.04**	**−0.497**
	**TGT (0.24)**	**0.02**	**0.212**
	**CAC (0.08)**	**0.005**	**−0.377**
	TAC (0.37)	0.16	0.109
	CGC (0.23)	0.97	−0.004
	**TGC (0.05)**	**0.02**	**−0.393**
**numCIG**	**CGT**	**0.0001** *	**−0.319**
	**TGT**	**0.02**	**0.068**
	CAC	0.06	−0.087
	TAC	0.18	0.035
	CGC	0.45	0.023
	**TGC**	**0.0002** *	**−0.212**
**FTND1**	CGT	0.19	−0.128
	TGT	0.92	−0.004
	CAC	0.81	−0.015
	TAC	0.05	0.070
	CGC	0.14	−0.061
	TGC	0.92	−0.007
**FTND5**	**CGT**	**1.21E-05** *	**0.63**
	**TGT**	**0.01**	**1.20**
	CAC	0.07	0.81
	TAC	0.21	1.08
	CGC	0.08	1.14
	**TGC**	**1.61E-05** *	**0.73**

Haplotypes are presented as rs919334-rs1457115-rs9285875. The frequency of each haplotype is in parenthesis next to the haplotype in the FTND phenotype row. Uncorrected significant p-values are bolded and underlined.

* denotes p-values that survived correction for multiple testing.

## Discussion

The goal of the current study was to elucidate the role of CaMKIV in nicotine dependence behaviors using both animal models and genetic association analysis. Results show that in CaMKIV −/− mice, nicotine CPP is attenuated, but cocaine CPP is enhanced. CaMKIV protein levels are also increased in the NAc after nicotine CPP. Further, anxiety-related behavior is not observed in CaMKIV −/− mice after precipitated nicotine withdrawal; however, somatic signs and the hyperalgesia response are not affected. We also identified variants and haplotypes in the promoter region of the CaMKIV gene associated with nicotine dependence. Findings from this study indicate the possibility of opposing roles for CaMKIV in nicotine and cocaine reward, and suggest that CaMKIV is involved in mediating affective, but not physical nicotine withdrawal behaviors. Significant association analyses further support the potential relevance of our findings to nicotine dependence in humans.

In the nicotine CPP assessment, the robust CPP observed in CaMKIV +/+ mice was significantly attenuated in CaMKIV −/− mice. CaMKIV protein levels were also increased in the NAc after nicotine CPP in C57Bl/6 mice. Similarly, pCREB levels were increased in the NAc and NAc shell after nicotine CPP [15], [16], while nicotine CPP was blocked by disruption of the CREB gene using CREB^αΔ^ mice [15] and by targeted disruption of CREB activation in the NAc shell [16]. CaMKIV phosphorylates CREB at Ser133 in the nuclei of neurons [9], [25]. While there are certainly other pathways that mediate CREB phosphorylation, such as extracellular regulated protein kinase, the current results suggest a possible contribution of the CaMKIV/CREB pathway in the NAc in processes involved in nicotine reward.

Conversely, cocaine CPP was enhanced in CaMKIV −/− mice, the opposite of that observed in the nicotine CPP assessment. These results are similar to those reported recently by Bilbaoa et al. [14] who showed that mice lacking CaMKIV in dopaminoceptive neurons displayed increased sensitivity to cocaine CPP. It is unlikely that differences in learning and memory attribute to these contrasting results, as CaMKIV −/− display normal spatial and learning memory [7]. Cocaine has been shown to regulate CREB in the NAc [10]. While overexpression of CREB in the NAc produced aversion to low doses of cocaine, overexpression of a dominant-negative mutant CREB increased the rewarding effects of cocaine [10]. Similar behavior was obeserved in CREB^αΔ^ which lack the major CREB isoforms [11]. Despite the studies implicating a connection in cocaine reward between CaMKIV and CREB in the NAc, inactivation of CaMKIV in dopaminoceptive neurons, including neurons in the NAc, enhanced cocaine CPP in a CREB-independent manner [14]. Thus, in the case of nicotine, while we cannot rule out the possible involvement of the CaMKIV/CREB pathway in nicotine reward, with cocaine, the involvement of CaMKIV appears to be independent of CREB, a finding that may contribute to the observed differences between the role of CaMKIV in nicotine and cocaine reward.

In the withdrawal assessment, the anxiety-related response was attenuated in CaMKIV −/− mice, but there was no significant difference between genotypes in either physical nicotine withdrawal measure, suggesting that CaMKIV has a role in mediating affective, but not physical nicotine withdrawal behaviors. While it is noted that paw tremors were significantly enhanced in CaMKIV −/− mice compared to +/+, the implications of which are unclear, these observations did not significantly impact the overall total average somatic signs, thus maintaining our hypothesis that CaMKIV is not involved in this aspect of nicotine withdrawal. Though we did not observe differences in anxiety-related behavior between vehicle treated CaMKIV +/+ and −/− mice, it has been reported that CaMKIV −/− mice exhibit decreased anxiety-like behavior based on the light/dark transition and elevated plus maze tests [7], [26]. These differences may be attributed to stress as a result of mini pump implantation surgery and subsequent saline mini pump infusion in our study, which may have impacted our results. Results may also be attributed to differences in the breeding generation of mice used between studies. Thus, our results in this assessment are interpreted with caution, as we cannot rule out the possible contribution of the anxiolytic-like phenotype observed in CaMKIV −/− mice.

Similar results were observed in a previous study from our lab with CaMKII +/− mice, where there was a loss of anxiety-related behavior, but physical withdrawal signs were present [27]. Interestingly, the current results are the opposite of that observed with the CaMKII inhibitor, KN-93, which was shown to enhance nicotine withdrawal-induced anxiety related behavior and block somatic signs [27]. KN93 blocks both CaMKII and CaMKIV activity [28], and it was suspected that the discrepancy between our previous CaMKII genetic and pharmacological data was due to KN-93 activity at CaMKIV [27]; however, the current results refute this notion. It is also possible that CaMKII +/− mice, which still possess 50% of the enzyme activity, do not have sufficient loss of activity to induce alterations that contribute to the observed behaviors after nicotine withdrawal. Further, these two enzymes may simply have differential involvement in mediating nicotine withdrawal behaviors.

Because affective nicotine withdrawal signs, which are hypothesized to contribute more to relapse than somatic signs [29], [30], were attenuated in CaMKIV −/− mice, these results suggest that CaMKIV levels are increased after withdrawal from nicotine, and reducing CaMKIV gene expression alleviates certain aspects of nicotine dependence. Indeed, pCREB and CREB levels were increased in the VTA and NAc respectively, after nicotine withdrawal [31]. While in the current study, we did not test the conditioned place aversion model, an additional affective measure of nicotine withdrawal, increased CREB activity in the NAc produces conditioned place aversion in mice [32]. CaMKIV in the NAc also modulates emotional behavior in mice [33]. As observed in our reward assessment, we propose that these studies support a role for CaMKIV in affective withdrawal measures, and taken together with previous reports, may implicate involvement of the CaMKIV/CREB pathway in the NAc.

Results from our human genetic association study support the animal data from our withdrawal assessment, implicating genetic variation in the CaMKIV gene promoter in nicotine dependence. Specifically, the rs919334 polymorphism is associated with a protective effect against nicotine dependence, based on lower FTND scores, while the rs9285875 polymorphism is associated with risk for higher FTND scores. While these findings initially appear to conflict, our multi-marker haplotype analysis was consistent with our single marker analysis, revealing that the presence of the minor alleles for both of these markers in combination (C-G-T) represents a significant protective haplotype against higher FTND scores, smoking a higher number of cigarettes per day, and smoking more cigarettes in the morning, while the T-G-T combination, consisting of the minor rs9285875 risk allele and the major risk alleles for the other two markers represents significant risk for higher FTND scores, smoking more cigarettes per day, and smoking more cigarettes in the morning. These results indicate that the haplotype analysis was more powerful in detecting significant associations with our smoking phenotypes than the single marker analysis, as we were able to detect significant associations with numCIG and FTND5 that were not evident in our single marker analysis, as well as obtain lower p-values that survived correction for multiple testing. Our genetic findings identify protective haplotypes that potentially reflect a reduced risk of developing nicotine dependence and a less severe withdrawal syndrome. Coinciding with our behavioral results, attenuated rewarding effects of nicotine and alleviation of affective nicotine withdrawal behaviors in CaMKIV −/− mice may also reflect protective mechanisms against development or maintenance of nicotine dependence. Further, because these variants are located in the promoter region of the CaMKIV gene, it is possible that they affect gene expression. Similar to the findings from our CPP assessments, these results are the opposite of that observed for cocaine, where rs919334 was associated with risk for cocaine addiction [14]. Interestingly, a similar effect was observed with the nonsynonymous polymorphism, rs16969968, located in the α5 nAChR gene, which is associated with risk for nicotine dependence, but protective against cocaine dependence [34]. The current results further support the hypothesis that such bidirectional associations between nicotine and cocaine dependence stem from the involvement of nAChRs in modulating both excitatory and inhibitory dopamine-mediated reward pathways [34], which may in turn differentially regulate downstream calcium-dependent processes. While additional studies are necessary to determine if these CaMKIV variants do indeed influence gene expression, we hypothesize that increased CaMKIV gene expression exerts a protective effect in nicotine dependence, but enhances the risk for cocaine dependence.

Our findings from the current study identify a calcium-dependent protein, a relevant brain region, and genetic haplotypes involved in nicotine dependence behaviors. Specifically, our studies support an important role for CaMKIV in mediating nicotine reward, and suggest that increased CaMKIV protein levels in the NAc influence this behavior. Our results further implicate a role for the protein in nicotine withdrawal behaviors. Further, we show that the findings in our reward study extend to cocaine, as alterations in the CaMKIV gene also mediate cocaine reward, though the role is the opposite of that observed with nicotine. Lastly, we support our studies with human genetic association, showing that variation in the CaMKIV gene is protective against nicotine dependence, a finding also opposite of that found in cocaine studies assessing CaMKIV gene variation. Overall, these studies further indicate the importance of calcium-dependent mechanisms in drug-mediated behaviors.

## References

[1] Mulle C, Choquet D, Korn H, Changeux JP (1992) Calcium influx through nicotinic receptor in rat central neurons: its relevance to cellular regulation. Neuron 8: 135–143. 1309647 10.1016/0896-6273(92)90115-t

[2] Lisman J, Schulman H, Cline H (2002) The molecular basis of CaMKII function in synaptic and behavioral memory. Nature 3: 175–190. 10.1038/nrn753 11994750

[3] Ohmstede CA, Jensen KF, Sahyoun NE (1989) Ca2+/calmodulin-dependent protein kinase enriched in cerebellar granule cells. Identification of a novel neuronal calmodulin-dependent protein kinase. J Biol Chem 264: 5866–5875. 2538431

[4] Jones DA, Glod J, Wilson-Shaw D, Hahn WE, Sikela JM (1991) cDNA sequence and differential expression of the mouse Ca2+/calmodulin-dependent protein kinase IV gene. FEBS Lett 289: 105–109. 1893997 10.1016/0014-5793(91)80919-t

[5] Frangakis MV, Chatila T, Wood ER, Sahyoun N (1991) Expression of a neuronal Ca2+/calmodulin-dependent protein kinase, CaM kinase-Gr, in rat thymus. J Biol Chem 266: 17592–17596. 1894642

[6] Jensen KF, Ohmstede CA, Fisher RS, Olin JK, Sahyoun N (1991) Acquisition and loss of a neuronal Ca2+/calmodulin-dependent protein kinase during neuronal differentiation. Proc Natl Acad Sci U S A 88: 4050–4053. 2023954 10.1073/pnas.88.9.4050 PMC51591

[7] Takao K, Tanda K, Nakamura K, Kasahara J, Nakao K, et al (2010) Comprehensive behavioral analysis of calcium/calmodulin-dependent protein kinase IV knockout mice. PLoS One 5: e9460. 20209163 10.1371/journal.pone.0009460 PMC2830479

[8] Deisseroth K, Heist EK, Tsien RW (1998) Translocation of calmodulin to the nucleus supports CREB phosphorylation in hippocampal neurons. Nature 392: 198–202. 9515967 10.1038/32448

[9] Soderling TR (1999) The Ca–calmodulin-dependent protein kinase cascade. Trends Biochem Sci 24: 232–236. 10366852 10.1016/s0968-0004(99)01383-3

[10] Carlezon WA Jr, Thome J, Olson VG, Lane-Ladd SB, Brodkin ES, et al (1998) Regulation of cocaine reward by CREB. Science 282: 2272–2275. 9856954 10.1126/science.282.5397.2272

[11] Walters CL, Blendy JA (2001) Different requirements for cAMP response element binding protein in positive and negative reinforcing properties of drugs of abuse. J Neurosci 21: 9438–9444. 11717377 10.1523/JNEUROSCI.21-23-09438.2001 PMC6763933

[12] Wei F, Qiu CS, Liauw J, Robinson DA, Ho N, et al (2002) Calcium calmodulin-dependent protein kinase IV is required for fear memory. Nat Neurosci 5: 573–579. 12006982 10.1038/nn0602-855

[13] Ko SW, Jia Y, Xu H, Yim SJ, Jang DH, et al (2006) Evidence for a role of CaMKIV in the development of opioid analgesic tolerance. Eur J Neurosci 23: 2158–2168. 16630062 10.1111/j.1460-9568.2006.04748.x

[14] Bilbao A, Parkitna JR, Engblom D, Perreau-Lenz S, Sanchis-Segura C, et al (2008) Loss of the Ca2+/calmodulin-dependent protein kinase type IV in dopaminoceptive neurons enhances behavioral effects of cocaine. Proc Natl Acad Sci U S A 105: 17549–17554. 19001277 10.1073/pnas.0803959105 PMC2582267

[15] Walters CL, Cleck JN, Kuo YC, Blendy JA (2005) Mu-opioid receptor and CREB activation are required for nicotine reward. Neuron 46: 933–943. 15953421 10.1016/j.neuron.2005.05.005

[16] Brunzell DH, Mineur YS, Neve RL, Picciotto MR (2009) Nucleus accumbens CREB activity is necessary for nicotine conditioned place preference. Neuropsychopharmacology 34: 1993–2001. 19212318 10.1038/npp.2009.11 PMC2709692

[17] Kivinummia T, Kastea K, Rantamäkib T, Castrénb E, Ahteea L (2011) Alterations in BDNF and phospho-CREB levels following chronic oral nicotine treatment and its withdrawal in dopaminergic brain areas of mice. Neurosci Lett 491: 108–112. 21232579 10.1016/j.neulet.2011.01.015

[18] Kota D, Martin BR, Robinson SE, Damaj MI (2007) Nicotine dependence and reward differ between adolescent and adult mice. J Pharmacol Exp Ther 322: 399–407. 17446302 10.1124/jpet.107.121616

[19] Paxinos G, Franklin K (2001) The Mouse Brain in Stereotaxic Coordinates, Second Edition. San Diego: Academic Press.

[20] Jackson KJ, Martin BR, Changeux JP, Damaj MI (2008) Differential role of nicotinic acetylcholine receptor subunits in physical and affective nicotine withdrawal signs. J Pharmacol Exp Ther 325: 302–312. 18184829 10.1124/jpet.107.132977 PMC3821841

[21] Purcell S, Neale B, Todd-Brown K, Thomas L, Ferreira MA, et al (2007) PLINK: a tool set for whole-genome association and population-based linkage analyses. Am J Hum Genet 81: 559–575. 17701901 10.1086/519795 PMC1950838

[22] Scheet P, Stephens M (2006) A fast and flexible statistical model for large-scale population genotype data: applications to inferring missing genotypes and haplotypic phase. Am J Hum Genet 78: 629–644. 16532393 10.1086/502802 PMC1424677

[23] Magi R, Morris AP (2010) GWAMA: software for genome-wide association meta-analysis. BMC Bioinformatics 11: 288. 20509871 10.1186/1471-2105-11-288 PMC2893603

[24] Price AL, Patterson NJ, Plenge RM, Weinblatt ME, Shadick NA, et al (2006) Principal components analysis corrects for stratification in genome-wide association studies. Nat Genet 38: 904–909. 16862161 10.1038/ng1847

[25] Deisseroth K, Tsien RW (2002) Dynamic multiphosphorylation passwords for activity-dependent gene expression. Neuron 34: 179–182. 11970860 10.1016/s0896-6273(02)00664-5

[26] Shum FW, Ko SW, Lee YS, Kaang BK, Zhuo M (2005) Genetic alteration of anxiety and stress-like behavior in mice lacking CaMKIV. Mol Pain 1: 22. 16102169 10.1186/1744-8069-1-22 PMC1208947

[27] Jackson KJ, Damaj MI (2009) L-type calcium channels and calcium/calmodulin-dependent kinase II differentially mediate behaviors associated with nicotine withdrawal in mice. J Pharmacol Exp Ther 330: 152–161. 19336664 10.1124/jpet.109.151530 PMC2700166

[28] Hook SS, Means AR (2001) Ca2_/CaM-dependent kinases: from activation to function. Annu Rev Pharmacol Toxicol 41: 471–505. 11264466 10.1146/annurev.pharmtox.41.1.471

[29] Koob GP, Heinrichs SC, Pich EM, Menzaghi F, Baldwin H, et al (1993) The role of corticotropin-releasing factor in behavioral responses to stress. Ciba Found Symp 172: 277–289. 8491090 10.1002/9780470514368.ch14

[30] Markou A, Kosten TR, Koob GF (1998) Neurobiological similarities in depression and drug dependence: A self-medication hypothesis. Neuropsychopharmacology 18: 135–174. 9471114 10.1016/S0893-133X(97)00113-9

[31] Brunzell DH, Russell DS, Picciotto MR (2003) In vivo nicotine treatment regulates mesocorticolimbic CREB and ERK signaling in C57Bl/6J mice. J Neurochem 84: 1431–1441. 12614343 10.1046/j.1471-4159.2003.01640.x

[32] Barrot M, Olivier JD, Perrotti LI, DiLeone RJ, Berton O, et al (2002) CREB activity in the nucleus accumbens shell controls gating of behavioral responses to emotional stimuli. Proc Natl Acad Sci USA 99: 11435–11440. 12165570 10.1073/pnas.172091899 PMC123274

[33] Schneider M, Spanagel R, Zhang SJ, Bading H, Klugmann M (2007) Adeno-associated virus (AAV)-mediated suppression of Ca2+/calmodulin kinase IV activity in the nucleus accumbens modulates emotional behaviour in mice. BMC Neurosci 8: 105. 18053176 10.1186/1471-2202-8-105 PMC2219998

[34] Grucza RA, Wang JC, Stitzel JA, Hinrichs AL, Saccone SF, et al (2008) A risk allele for nicotine dependence in CHRNA5 is a protective allele for cocaine dependence. Biol Psychiatry 64: 922–929. 18519132 10.1016/j.biopsych.2008.04.018 PMC2582594

